# Black adults show cross-sectional associations between sleep quality and episodic memory retention: an investigation of underlying factors

**DOI:** 10.3389/frsle.2026.1968296

**Published:** 2026-10-14

**Authors:** Emily Hokett, Jess Campbell, Aditi Arunmozhi, Audrey Duarte

**Affiliations:** 1 Department of Psychology, Florida Atlantic University, Boca Raton, FL, United States; 2 Department of Neurology, University of Texas at Austin, Austin, TX, United States; 3 Department of Psychology, Georgia Institute of Technology, Atlanta, GA, United States; 4 Department of Psychology, University of Texas at Austin, Austin, TX, United States

**Keywords:** depression, episodic memory, ethnicity, memory retention, race, sleep quality

## Abstract

Non-Hispanic Black adults have poorer sleep quality than non-Hispanic White adults (hereafter, Black, White), which may underlie disparities in cognition. Epidemiological evidence suggests episodic memory may be more sensitive to poor sleep quality in older Black adults as compared to older White adults. Related exacerbating factors of these differences, such as depression and discrimination, are understudied. Here, we aimed to determine mediators of associations between sleep quality and episodic memory retention in an adult lifespan sample (18–74 years) of Black and White adults. We measured sleep quality, depression, and discrimination using self-report questionnaires, and assessed memory retention using a context memory task. We assessed if sleep-memory associations were moderated by age and mediated by depressive symptoms. We also assessed if discrimination mediated the association sleep-memory associations, controlling for age and years of education. In Black and White adults, higher depressive symptoms were associated with poorer sleep quality. Black adults demonstrated reliable sleep-memory associations and associations among higher discrimination, higher depressive symptoms, poorer sleep, and poorer memory, but we found no significant mediation effects. Depression level might affect sleep quality regardless of race or age. Black adults may demonstrate poor memory retention because of associations among discrimination, depression, and sleep.

## 1 Introduction

Sleep is important for the consolidation of episodic memory (e.g., memory for past events or episodes) (Rasch and Born, 2013). Using standardized neuropsychological tests, epidemiological research has shown lower episodic memory performance in older non-Hispanic Black adults as compared to older non-Hispanic White adults (hereafter, Black, White) (Chen et al., 2022; Zahodne et al., 2016). To reduce racial and ethnic disparities in cognitive performance, it is important to identify modifiable factors that may contribute to these cognitive disparities. One such modifiable factor is sleep quality.

Older adults, as compared to young and middle-aged adults, are more likely to experience chronic sleep disruptions that include reduced sleep duration and decreased sleep continuity (Ohayon et al., 2004). Poorer sleep quality has been associated with poorer episodic memory performance using self-report, actigraphy, and polysomnography-measured sleep in young and older adults (Hokett et al., 2021). Greater self-reported nighttime awakenings and higher actigraphy-measured wake after sleep onset (WASO) have been associated with poorer recall performance in older adults (Mary et al., 2013; Wilckens et al., 2014). However, these findings may be primarily based on data from White adults, as demographic information was omitted from most studies. Sleep disparities between Black and White adults have been well documented (Johnson et al., 2019). Black adults often experience shorter sleep duration and poorer sleep quality (e.g., lower slow wave sleep density, lower self-reported sleep quality) than White adults (Johnson et al., 2019). However, the impact of poor sleep on episodic memory performance is unclear in minoritized racial and ethnic groups across the adult lifespan, and potential interactions with age-related declines in sleep quality and memory performance are understudied.

## 2 Background

Only a few observational studies have assessed associations between sleep quality and episodic memory in Black adults. Episodic memory performance in older Black, compared to older White, adults may be particularly sensitive to the negative effects of poor sleep quality (Owusu et al., 2022; Swanson et al., 2021). The higher variation in habitual, non-manipulated sleep quality in Black adults may also contribute to associations between sleep and memory (Hokett et al., 2026b). Importantly, these studies used standardized, neuropsychological memory tests, specifically word lists, which do not involve contextually binding associations. Differing from these studies, experimental memory task designs can vary demands on cognitive control, and sleep may be particularly beneficial for tasks that place high demands on cognitive control functions, such as relational integration, recovering specific episodic features in the presence of distractors, and interference resolution (Diekelmann et al., 2009). These experimental memory tasks require deliberate, top-down processing that is mediated by prefrontal function (Duarte and Dulas, 2020). Retroactive interference tasks involve remembering old information while ignoring recently presented, new information, which requires high cognitive control. Retroactive interference studies have also shown greater sleep benefits for memory performance as compared to tasks with no interference (Ellenbogen et al., 2009; Sonni and Spencer, 2015). Collectively, these studies demonstrate that sleep may be more beneficial for memory performance for tasks that require greater cognitive control. The present study examines associations between sleep quality and episodic memory performance requiring high cognitive control, namely delayed associative memory retention for image pairs. To place higher cognitive control demands on delayed memory retrieval, the delayed memory task was immediately preceded by a retroactive interference task.

In our previous research examining sleep-memory associations, Black adults, across young and older age, demonstrated stronger associations between poorer sleep quality and lower neural activity supporting episodic memory retrieval than did White adults (Hokett and Duarte, 2019). Similarly, adults from minoritized racial and ethnic groups, primarily Black adults, showed stronger associations between poorer sleep quality and familiarity-based similarity in neural activity between encoding and retrieval (Hokett et al., 2022). Given that familiarity-based recognition memory lacks contextual, episodic details, we hypothesized that these results suggest poorer sleep quality is linked with greater reliance on familiarity-based recognition (i.e., lacking episodic integrity) in minoritized racial and ethnic groups. Due to relatively small sample sizes in these previous studies, we were underpowered to explore factors that may underlie racial and ethnic differences in these associations. Negative factors, including discrimination, may impact racial and ethnic differences in sleep-memory associations. More distinct events of discrimination and chronic, high frequency, experiences with discrimination may lead to high stress and poor cognitive performance (Forrester et al., 2019). Racial discrimination contributes to poor sleep quality (Slopen and Williams, 2014; Hokett et al., 2026a), but previous studies have not assessed mediating effects of discrimination on sleep-memory associations.

Depression may also underlie sleep-memory associations. Depressive symptoms have been independently associated with both poor sleep quality and poor memory performance (Fang et al., 2019; James et al., 2021). Sleep disturbance is considered a symptom of depression, and longitudinal evidence suggests sleep disorders may predict depression onset (Fang et al., 2019). The association between sleep quality and subjective cognitive decline, including subjective memory complaints, may be partially explained by depressive symptoms (Xu et al., 2021). Depression is also associated with lower hippocampal volume, which may contribute to poor episodic memory performance (James et al., 2021). People who experience higher discrimination also experience higher depressive symptoms (Williams, 2018). Given that Black adults disproportionately experience discrimination, poor sleep quality, and poorer cognitive outcomes (Zahodne et al., 2016; Johnson et al., 2019; Slopen et al., 2016), further assessment of these factors in racially and ethnically diverse groups that include Black adults is warranted to address if sleep and memory associations are explained by either depressive symptoms or discrimination.

The present study aimed to assess sleep-memory associations in Black and White adults across the adult lifespan and identify specific factors that may contribute to associations between sleep quality and episodic memory performance within Black adults. Given recent epidemiological findings demonstrating that Black adults may show stronger associations between sleep quality and episodic memory performance than White adults (Hokett and Duarte, 2024), we aimed to assess within-group associations between sleep quality and memory performance using an index of sleep-dependent memory consolidation: associative episodic memory retention (i.e., differences in memory performance between immediate and delayed retrieval). Sleep (as compared to wake) has been associated with such measures of episodic memory consolidation, although sleep-based memory consolidation may be dampened with older age (Rasch and Born, 2013; Berres and Erdfelder, 2021; Scullin and Bliwise, 2015). A recent meta-analysis demonstrated episodic memory retention is more sensitive to sleep than other memory measurements (Berres and Erdfelder, 2021). Our experimental measure of episodic memory retention comprehensively assesses controlled memory performance, such as details related to the associative context of a memory (e.g., discriminating between previously seen face-object pairs and recombined face-object pairs). Moreover, we administered an interference task to increase the level of cognitive control required to perform the delayed associative memory task. These experimental memory tasks are sensitive to sleep (Diekelmann et al., 2009; Ellenbogen et al., 2009) and age (Duarte and Dulas, 2020). Given previous research that age may moderate the association between sleep quality and episodic memory performance (Wilckens et al., 2014; Hokett and Duarte, 2019; Hokett et al., 2022), we assessed age as a moderator for sleep and memory retention associations. To assess the generalizability of associations between sleep quality and episodic memory performance, we separately assessed analyses within each racial and ethnic group (Black, White), which allows us to capture associations that may be missed by only assessing significant interaction effects (Ward et al., 2019). We also hypothesized Black adults across the adult lifespan, including middle-age, would demonstrate reliable sleep-memory associations (Owusu et al., 2022; Swanson et al., 2021), and the association between sleep quality and memory performance may be mediated by depressive symptoms (Xu et al., 2021). Moreover, we examined the mediating role of discrimination on sleep-memory associations in Black adults.

## 3 Materials and methods

### 3.1 Race and ethnicity

In this study, we assess race and ethnicity to understand the ongoing effects of racialization and racially patterned psychosocial stress (e.g., discrimination, depression) on sleep quality and episodic memory retention. Participants in the present analysis were categorized as Black (non-Hispanic Black), and White (non-Hispanic White) according to their self-selected racial and ethnic categorization.

### 3.2 Participants

We recruited participants online using Prolific (https://www.prolific.co/). All participants were U.S. residents with sufficient eyesight to see a computer screen and proficient in the English language. The present participant sample is a subsample of a previously published study in which memory was not investigated (Hokett et al., 2022a). We excluded individuals who self-reported neurological disease (e.g., cerebrovascular disease, Parkinson's disease, seizures). Two hundred seventy-eight participants completed all tasks required to measure episodic memory retention. Of the 278, we excluded one participant with an extreme value for memory retention (3 SD higher than the mean) and 63 participants who did not complete all questionnaires of interest (questionnaires explained below). Our analytic sample included 214 adults (56 Black and 158 White adults; see Table 1) with complete data on all variables of interest. This study was approved by the Georgia Institute of Technology Institutional Review Board, as all authors were formerly affiliated with this institution. All participants completed informed consent forms.

**Table 1:** Participant demographics.

Participant characteristics	Black (*n* = 56)	White (*n* = 158)
Age	36.5 (11.0)	43.9 (16.6)
Women	40 (71.4%)	94 (59.5%)
Education (Years)	16.3 (2.12)	15.5 (2.39)
Sleep quality	5.68 (3.89)	7.01 (3.85)
Memory retention (*d′*)	−0.96 (0.69)	−1.07 (0.68)
Immediate memory (*d′*)	1.73 (0.92)	1.92 (0.93)
Delayed MEMORY (*d′*)	0.77 (0.57)	0.85 (0.51)
Delayed non-interference memory (*d′*)	0.76 (0.61)	0.87 (0.68)
Delayed interference memory (*d′*)	0.82 (0.71)	0.91 (0.63)
Depressive symptoms	3.48 (5.11)	5.23 (5.54)
Discrimination events	5.36 (2.28)	2.56 (1.09)
Discrimination frequency	14.4 (8.71)	4.72 (3.96)

Mean (SD); *n* (%) for Women. Memory retention (*d*′) is the difference between immediate memory (*d*′) and delayed memory (*d*′), and smaller negative values indicate lower decay, or better memory retention.

### 3.3 Materials

#### 3.3.1 Questionnaires

All questionnaire items are included in Appendix A. We provide a summary of each questionnaire below.

#### 3.3.2 Sleep quality

We measured global sleep quality with the Pittsburgh Sleep Quality Index (PSQI) (Buysse et al., 1989). This measure assesses several difficulties with getting to sleep and staying asleep throughout the night (e.g., uncomfortable temperature, snoring, pain) over the prior month. Higher scores on the PSQI reflect poorer sleep quality (range: 0–21).

#### 3.3.3 Depression

We measured depressive symptoms (range: 0–21) using the Depression subscale of the Depression, Anxiety, and Stress Scale-21 (DASS-21) (Lovibond and Lovibond, 1995).

#### 3.3.4 Self-reported discrimination

To assess self-reported discrimination, we measured the number of discrimination events (range: 0–9) and frequency of discrimination (range: 0–45) with the Experiences of Discrimination Scale (EOD) (Krieger et al., 2005). The EOD requires participants to determine if they have experienced events of discrimination related to their race, ethnicity, or skin color. More events represent more varied experiences with discrimination. More encounters represent higher discrimination frequency. To give all participants the same testing protocol, this measure was administered to all participants. However, we only assess discrimination in Black adults to better understand factors that may contribute to disparities in sleep quality and memory performance.

#### 3.3.5 Images: context faces and scenes and objects

Color item images (64), context faces (8), and context scenes (8) were collected from visual object databases used in prior studies (e.g., the Bank of Standardized Stimuli) (Brady et al., 2008; Brodeur et al., 2010), Google images, and Unsplash. The images consisted of either natural or manmade objects (e.g., well, apple, wallet, water bottle). Eight context face images were evenly paired across object images. Specifically, we divided across race (Black, White), age (young, old), and gender (woman, man). This included a young Black woman and man, a young White woman and man, an older Black woman and man, and an older White woman and man. Also, eight context scenes were balanced across outdoor (forest, city, ocean, mountain) and indoor (office, house, classroom, diner) categories. See Appendix B for all context images.

### 3.6 Design and procedure

We recruited participants age 18 to 80 years, and our analytic sample ranged from age 18 to 74 years. Participants were asked to complete two separate experimental sessions over two days. The tasks were administered using online platforms: Qualtrics and Pavlovia. Data was collected from around March to June 2021. Participants were informed that they needed to complete both sessions to receive compensation unless they experienced extenuating circumstances (e.g., illness, internet outage). See Figure 1 for a depiction of the experimental design.

**Figure 1:**
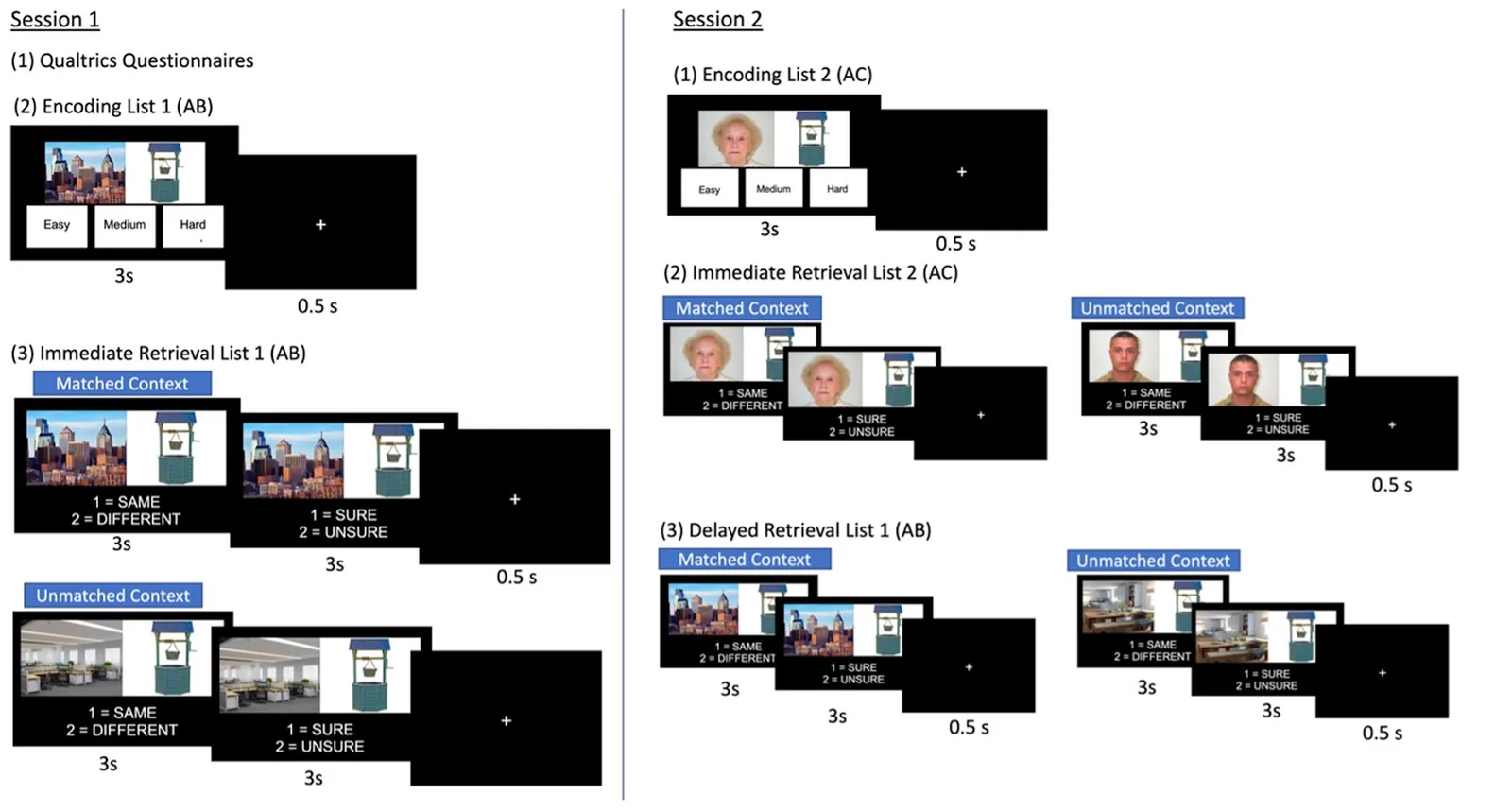
Experimental design for sessions 1 and 2.

#### 3.6.1 Session 1: encoding and immediate retrieval

In Session 1, participants completed questionnaires on Qualtrics (see Section 2.3.1). Then, they were redirected to Pavlovia for the first experimental task. Before encoding, participants viewed an instructional video and were given a practice encoding task with 40 image pairs. We used a high number of practice trials to ensure participants understood the task. The experimental encoding task involved rating a series of 64 object-face or object-scene image pairs by clicking keys corresponding to Easy, Medium, or Hard based on their subjective ability to imagine the two items together in a natural environment. For example, a participant might report finding a city and well, as shown in Figure 1, difficult to imagine paired together in a real-world setting. Similarly, participants were presented with face-object pairs. A participant might be able to easily imagine a person drinking from a water bottle but less easily imagine a person interacting with a killer whale. This orienting task was designed to facilitate deep encoding of object-context associations. All encoded pairs in Session 1 are AB pairs. Each image pairing included one A/natural object and one B/context image (face or scene; see Section 2.3.2). Each object was only presented once during each task. Participants were given 3 s for each response. We administered multiple versions of the task to counterbalance the objects for trial type (matched/unmatched) and image presentation (left or right side).

Following encoding, we instructed participants to complete an immediate retrieval task for the AB pairs. As with encoding, participants were given 40 practice retrieval pairs. During the experimental retrieval task, we represented participants with 64 image pairs from encoding. Half of the retrieval pairs were paired in the same way as at encoding (e.g., matched context shown in Figure 1), and the other half were paired differently from encoding (e.g., unmatched context shown in Figure 1). For the unmatched pairs that were different from encoding, the context was switched within the same context category (e.g., scene context will be switched with another scene), but it was never in the nearest context category (e.g., outdoor scenes, such as a city, were never switched with another outdoor scene; it was switched to an indoor scene, e.g., an office). This method was chosen to maintain a moderate level of task difficulty, as mismatched pairs tend to be more effortful to reject as mismatched than intact pairs are to recognize as intact. Participants determined if the image pairs matched or did not match those presented during encoding. Immediately following this response, they were asked to rate their confidence in their decision. Participants were allowed 3 s for each retrieval response.

#### 3.6.2 Session 2: interference memory task and delayed retrieval

We used the interference memory task to place higher cognitive control demands on participants when completing the delayed memory task (explained below). Participants were required to complete Session 2 after a 24 to 36-h interval, depending on their schedules, for convenience, which is in alignment with previous memory retention studies (Ritchey et al., 2015; Scullin, 2013). Procedures for Session 2 were nearly identical to those in Session 1. At Session 2, participants completed a new encoding and immediate retrieval interference task with 32 interference pairs (indicated as AC in Figure 1). These AC pairs were composed of half of the studied objects from Session 1 presented with a different context category from that of the Session 1 encoding session. For example, if the original pair was a city scene (context) and a previously studied well (object), the interference pair was either a male or female face (context) and well (object). The encoding procedures were identical to those of Session 1.

Following the interference encoding task, participants were given an immediate retrieval task for the AC pairs. The immediate retrieval procedures were identical to those of retrieval at Session 1. However, participants were given explicit instructions that their memory decisions should be based on the encoding task for the current day. Immediately following the interference encoding and immediate retrieval tasks, participants were given a delayed retrieval test that involved remembering images from Session 1 only (indicated as AB pairs in Figure 1). An instructional video preceded the delayed retrieval task to ask participants to respond based on the original items from Session 1, not the items presented during the present day (i.e., Session 2).

## 4 Memory performance and data quality

To increase the degree of cognitive control required to retrieve image pairs, we placed an interference memory task immediately before the delayed memory task. Our outcome measure of interest was memory retention using *d'*. To calculate each *d'* measure, we used the standard formula (i.e., standardized hits–false alarms) (Stanislaw and Todorov, 1999). Specifically, hits are previously encoded pairs correctly recognized as matching those presented during encoding (matched recognized as matched) and false alarms are unmatched pairings that are incorrectly identified as previous pairings (unmatched selected as matched). We calculated memory retention as the difference between *d'* at delayed retrieval and *d'* at immediate retrieval for AB pairs. Memory decay is expected, and smaller negative values indicate lower memory decay (better memory performance).

Interference memory performance was measured at delayed retrieval to determine if introducing interference lowers memory retention performance for the original AB pairs. We include memory performance for these pairs in the interference condition in Table 1. To retain most of our sample while ensuring data quality, we included participants with no more than 20% of absent responses on each memory task. Thus, some participants with absent responses had slightly different numbers of trials for each condition, and delayed memory (across conditions) is not an exact average between interference and non-interference conditions.

### 4.1 Statistical analysis

We used paired *t*-tests to examine the difference between the interference and non-interference conditions. To assess effects of racialization and racial discrimination, we stratified the sample by racial and ethnic group (Hokett et al., 2026b; Ward et al., 2019). We examined associations between sleep quality and memory retention. We also tested age as a continuous moderator of the association between sleep quality and memory retention. All regression tables were generated using the stargazer package in R (Sjoberg et al., 2021).

We examined depressive symptoms as a mediator of the association between sleep quality and memory retention. We also assessed self-reported discrimination (discriminatory events and frequency, separately) as a mediator of the sleep-memory association in Black adults. We separately considered these mediators. All mediation analyses were performed in R using the mediation package (Tingley et al., 2014). In tables, we reported the average causal mediation effect (i.e., indirect effect), average direct effect, and total effect with 95% confidence intervals. Mediation effects were estimated with bootstrapped confidence intervals using 500 simulations.

In each analysis, we controlled for years of education and (when not assessing age moderation effects) age. Education may influence cognitive performance and is typically included as a covariate in sleep and memory analyses (Hokett et al., 2026b), and older age is associated with poorer sleep quality and memory (Ohayon et al., 2004; Duarte and Dulas, 2020). We defined race and ethnicity as a categorical variable and stratified all analyses by racial and ethnic group (Black, White). All other factors were assessed as continuous variables. Statistical analyses were conducted using the statistical package of social sciences (SPSS) 27 and R Version 2024.04.2+764.

## 5 Results

Memory retention for non-interference pairs was lower than memory performance for interference pairs, but these differences were not statistically significant across interference conditions (see Table 1; *t*_213_ = 0.81, *p* = 0.21). Because we found no reliable differences between memory retention for interference pairs as compared to non-interference conditions, we collapsed across interference conditions for subsequent analyses.

### 5.1 Black, but not White, adults demonstrated sleep-memory associations

Stratified by racial and ethnic group and controlling for years of age and years of education, we assessed the association between sleep quality and memory retention. Black adults showed significant associations between poorer sleep quality and poorer memory performance (i.e., lower retention scores), but not White adults (See Figure 2 and Table 2).

**Figure 2:**
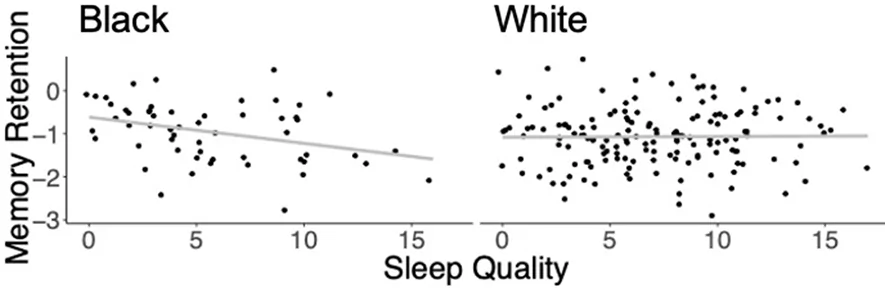
Black adults (*n* = 56) demonstrated sleep-memory associations, but White adults (*n* = 158) demonstrated no reliable association. Higher memory retention (i.e., Delayed Memory d'–Immediate Memory d') values reflect higher retention; lower (more negative) values represent lower retention. Higher values of sleep quality are reflective of poorer sleep quality (i.e., more complaints), and lower values are reflective of higher sleep quality (i.e., fewer complaints).

**Table 2:** Beta estimates for associations with memory retention in Black and White adults.

Beta estimates	Black adults (*n* = 56)	White adults (*n* = 158)
Sleep quality	−0.06^*^ (0.023)	0.003 (0.014)
	*p* = 0.014^*^	*p* = 0.853
Education	0.025 (0.043)	0.004 (0.023)
	*p* = 0.569	*p* = 0.871
Age	−0.002 (0.008)	0.002 (0.003)
	*p* = 0.855	*p* = 0.617

Beta estimate (standard error).

^*^p < 0.05.

### 5.2 Depressive symptoms did not explain the association between sleep quality and memory retention

We examined depressive symptoms as a mediator for the association between sleep quality and memory retention. Depressive symptoms did not reliably mediate sleep-memory associations in Black or White adults (see Supplementary Tables 1, 2). Regression analyses in both Black (*B* = 0.66, *p* < 0.001), and White (*B* = 0.63, *p* < 0.001) adults showed significant associations between poorer sleep quality and higher depressive symptoms.

### 5.3 Self-reported discrimination was associated with sleep quality and memory retention in Black adults

We further examined sleep-memory associations in Black adults by assessing self-reported experiences of discrimination. Specifically, we assessed if discrimination explained the association between sleep quality and memory retention in Black adults. Moreover, we tested if higher discrimination was associated with lower sleep quality, lower memory retention, and higher depressive symptoms.

We found no significant indirect effect for discrimination events (see Supplementary Table 3) or the frequency of discrimination (see Supplementary Table 4) for the association between sleep quality and memory retention. To assess if discrimination was associated with sleep quality, depressive symptoms, or memory retention, we ran separate linear regression models, controlling for years of age and years of education. Black adults showed associations between higher experiences of discrimination with higher depressive symptoms, lower memory retention, and poorer sleep quality (see Table 3).

**Table 3:** Beta estimates for associations between discrimination events and discrimination frequency with sleep quality, depressive symptoms, and memory retention in Black adults.

Beta estimates	Sleep quality	Depressive symptoms	Memory retention
Model with discrimination events as the predictor of interest			
Discrimination events	0.585^*^ (0.246)	1.030^*^ (0.303)	−0.051 (0.046)
	*p* = 0.022^*^	*p* = 0.002^*^	*p* = 0.267
Age	−0.013 (0.047)	−0.020 (0.058)	−0.001 (0.009)
	*p* = 0.791	*p* = 0.738	*p* = 0.894
Education	−0.459 (0.267)	−0.902^*^ (0.33)	0.060 (0.050)
	*p* = 0.093	*p* = 0.009^*^	*p* = 0.236
Model with discrimination frequency as the predictor of interest			
Discrimination frequency	0.149^*^(0.060)	0.248^*^ (0.074)	−0.023^*^ (0.011)
	*p* = 0.016^**^	*p* = 0.002^*^	*p* = 0.040^*^
Age	−0.009 (0.047)	−0.016 (0.059)	−0.003 (0.009)
	*p* = 0.843	*p* = 0.791	*p* = 0.764
Education	−0.322 (0.247)	−0.649^*^ (0.307)	0.056 (0.045)
	*p* = 0.198	*p* = 0.040^*^	*p* = 0.215

Beta estimate (standard error).

^*^p < 0.05; *n* = 56.

### 5.4 Age did not moderate sleep-memory associations

We found no moderating effects of age on associations between sleep quality and memory in either Black or White adults (see Table 4). In the **Discussion**, we speculate on possible explanations for this null finding.

**Table 4:** Beta estimates for interaction effect of sleep quality and age on memory retention in Black and White adults.

Beta estimates	Black adults (*n* = 56)	White adults (*n* = 158)
Sleep quality	−0.021 (0.096)	−0.007 (0.041)
	*p* = 0.825	*p* = 0.869
Age	0.005 (0.017)	0.0002 (0.007)
	*p* = 0.788	*p* = 0.971
Sleep quality x age	−0.001 (0.003)	0.0002 (0.001)
	*p* = 0.682	*p* = 0.806
Education	0.026 (0.044)	0.003 (0.023)
	*p* = 0.559	*p* = 0.885

Beta estimate (standard error).

## 6 Discussion

The present study is the first to measure associations between sleep quality and associative memory retention in Black and White adults, an aspect of episodic memory that is robustly linked to sleep (Berres and Erdfelder, 2021). Moreover, this is the first study to identity potential mechanisms explaining stronger associations between sleep quality and memory performance in Black adults as compared to White adults. Specifically, our findings suggest experiences of discrimination in Black adults across the lifecourse are associated with poorer sleep quality and higher depressive symptoms that may result in poorer associative memory retention. However, neither depression nor discrimination were reliable mediators of the association between sleep quality and memory retention. Our results suggest links among discrimination, poorer sleep quality, and poorer memory performance in Black adults, but more research is needed to better understand racial and ethnic differences in associations between habitual sleep quality and episodic memory retention.

### 6.1 Black but not White adults show sleep-memory associations

In alignment with previous epidemiological research (Owusu et al., 2022; Swanson et al., 2021), memory performance in Black adults was more sensitive to sleep quality than in White adults. For example, older Black adults showed associations between poorer sleep quality and poorer episodic memory performance (Owusu et al., 2022; Swanson et al., 2021) and memory-related neural oscillations (Hokett and Duarte, 2019) with actigraphy-measured sleep metrics. This study is the first experimental study to demonstrate that these associations are also observed with self-reported sleep quality and memory retention, which suggests associative memory retention is more sensitive to sleep quality in Black adults than White adults. In other words, the present study demonstrates episodic memory tasks that require high cognitive control (e.g., memory retention following an interference memory task) are reliably associated with habitual sleep quality in Black adults. Specifically, participants completed an interference memory task immediately before the delayed retrieval task. Given that participants must ignore recent pairings from the interference task and remember the initial items presented within the prior 24 to 36 h, our memory retention metric indexes highly controlled memory performance.

Differing from previous research only assessing racial and ethnic differences in sleep-memory associations (Owusu et al., 2022; Swanson et al., 2021), the present study aimed to examine racially patterned stressors, including self-reported discrimination and related depressive symptoms, that may exacerbate the association between poorer sleep quality and poorer memory retention in Black adults. Here, we extended this research by assessing self-reported discrimination and depressive symptoms as factors that may underlie these stronger associations between sleep and memory performance in Black adults.

Higher self-reported discrimination in Black adults was associated with poorer sleep quality, higher depressive symptoms, and poorer memory retention. We speculate that discrimination and depression may contribute to strong associations between poorer sleep quality and poorer memory retention in Black adults. Given that discrimination is associated with both poor sleep quality and depression (Slopen and Williams, 2014; Williams, 2018; Johnson et al., 2021), discriminatory experiences may drive sleep-memory associations in Black adults, who are disproportionately affected by discrimination. Although this is the first experimental study to investigate psychosocial factors in relation to racial and ethnic differences in sleep-memory associations, previous research has shown discrimination and related psychosocial factors may underlie the racial and ethnic disparities in cognition (Chen et al., 2022; Zahodne et al., 2019). One epidemiology study including Black and White participants from the Midlife Development in the United States Study (MIDUS) and the MIDUS Refresher Study found that cumulative stress partially explained disparities in executive function and episodic memory performance. Specifically, Black adults performed worse on these cognitive tests due, in part, to higher cumulative stress, involving childhood stress, neighborhood stress, financial stress, and discrimination. Similarly, in the nationally representative Health and Retirement Study, Black adults who experienced higher levels of discrimination also demonstrated poorer episodic memory, and greater discrimination in Black adults was associated with faster memory decline over six years (Zahodne et al., 2019). Our previous research has also shown racism-related vigilance, or anticipating racist events without their direct occurrence, is associated with poorer sleep quality in Black adults (Hokett et al., 2026a). Although speculative, such experiences with discrimination and racism-related vigilance might lead to intrusive, task-irrelevant thoughts that interfere with successful memory encoding and retrieval, which is an important hypothesis to test in future research. Taken together, depression, poor sleep quality, and discrimination are interrelated and may influence stronger sleep-memory associations in Black adults than White adults.

### 6.2 Age did not moderate the association between sleep quality and memory retention

The literature on age-related differences in sleep-memory associations is mixed. Some have posited that older adults may not show strong associations between sleep quality and memory performance because of age-related decline in brain structures that support sleep-based memory consolidation (i.e., functional weakening) (Scullin and Bliwise, 2015). Alternatively, memory performance in older adults, but not young adults, may be sensitive to specific types of sleep measurement, such as actigraphy-measured sleep quality (Hokett et al., 2021; Hokett and Duarte, 2019). By contrast, age differences in these associations are not commonly observed for self-reported sleep quality measures (Hokett et al., 2021). Perhaps underreporting sleep disturbances, such as night-time awakening, masks age-related differences in sleep-memory associations that are often detected with objective sleep measures. Notably, most prior research studies on sleep-memory associations were conducted in-person and may not be susceptible to the same biases as online-based studies. Participants in the present study may show no meaningful age differences in associations between sleep quality and memory retention because online participant recruitment might result in samples with higher socioeconomic status, including more formal education. Young and older adults recruited from online platforms may have higher access to resources and similar cognitive and sleep quality profiles as compared to young and older adults recruited from community-based samples. Future research should replicate this study in-person with objective measures of sleep quality and participants recruited from the community.

### 6.3 Strengths and limitations

Our study has several strengths. First, this study includes an adult lifespan sample, which allowed us to investigate age as a continuous moderator for associations between sleep quality and episodic memory performance (Hokett et al., 2022). Moreover, as Black adults make up approximately 14% of the United States population, we oversampled Black adults (26% of the present study) to improve the racial and ethnic diversity within our study. Second, we used an experimental associative memory retention task to assess a measure of episodic memory that requires high cognitive control and is sensitive to sleep (Duarte and Dulas, 2020; Berres and Erdfelder, 2021). This method differs from most previous epidemiological research that has assessed race and ethnicity in relation to sleep-memory associations using standardized neuropsychological memory tests (Owusu et al., 2022; Swanson et al., 2021). Such standardized assessments may not capture memory for episodic details (e.g., associative context or the setting in which an event occurred), which is measured with the experimental design used in the present study.

This study is not without limitations. We recruited all participants using an online platform. People with computer and Internet access in their homes may not represent the socioeconomic status of the general population, especially not older adults. Given the small sample size, we did not further analyze the results by gender, and previous research has found associations between sleep and memory recall, when stratified by gender and race and ethnicity, may vary by neighborhood conditions (Hokett and Duarte, 2024). Future research should further investigate if sleep and long-term memory retention interact with neighborhood conditions in women and men within different racial and ethnic groups. Moreover, although we instructed participants to perform the task in an environment where they could hear sound for instructional videos, participants may be more open to distractions when completing an online study as compared to one in a controlled laboratory setting. Future online research may request to record sound in participants' external environments and perform sensitivity analyses to understand if background sound affects memory performance in online studies. Our study is also limited by self-reported sleep metrics, which are subject to recall bias and inaccuracies as compared to objective sleep measurements (Jackson et al., 2020). Collecting both subjective and objective sleep measures in future studies could allow for more comprehensive assessments of sleep quality. Although we oversampled Black adults, our overall sample size was small and may not have been able to detect small effect sizes. The present study is cross-sectional and cannot determine directionality. Depression and sleep quality are bidirectionally associated (Fang et al., 2019) and thus necessitate longitudinal models to fully examine the causal pathway among sleep, depression, and memory.

## 7 Conclusion

The present study is novel, as we measured the association between sleep quality and a measure of memory performance that requires high cognitive control, associative memory retention, in an adult lifespan sample of Black and White adults. We observed associations between poorer sleep quality and poorer memory retention in Black adults, but not White adults. Importantly, we examined contributing factors to sleep-memory associations in Black adults, namely discrimination and depression.

Black and White adults showed associations between higher depressive symptoms and poorer sleep quality. Future research in larger samples should test if depression related to discrimination underlies differences in associations between sleep quality and memory retention in Black adults. Greater feelings of sadness and hopelessness from more experiences with discrimination may strengthen the association between poorer sleep quality and poorer memory retention in Black adults. Chronic experiences with discrimination and depression may contribute to lower hippocampal volume and may impact hippocampal-based memory consolidation and retrieval (Forrester et al., 2019). Future studies should assess underlying neuroimaging measures that mediate sleep-memory associations. Sleep research should also inform policy changes to reduce discriminatory experiences and assess if such changes lead to improvements in brain health, mental health, sleep health, and cognitive performance that could narrow racial and ethnic health disparities.

## Data Availability

The datasets presented in this article are not readily available because the authors collect sensitive information on participant demographics and need to ensure that the data will be properly handled. The raw data will be available upon reasonable request to the corresponding author. Requests to access the datasets should be directed to Emily Hokett, ehokett@fau.edu.
