# Supplementary material for: Black adults show cross-sectional associations between sleep quality and episodic memory retention: an investigation of underlying factors
Source: Front Sleep. 2026 Oct 14;5:1968296. doi: 10.3389/frsle.2026.1968296 (PMC13713361; doi:10.3389/frsle.2026.1968296)
Supplement: Supplementary file 1 [file Supplementary_file_1.docx]

Supplementary Material

**Appendix A. Questionnaires**

**Sleep Quality** (Pittsburgh Sleep Quality Index)^1^

Response options for items 1-4 were write-in responses. Responses for items 5a-j and 6-8 included: Not during the past month (0), Less than once a week (1), Once or twice a week (2), Three or more times week (3). Responses for item 9 were: Very good (0), Fairly good (1), Fairly bad (2), Very bad (3).

1. When have you usually gone to bed? ______________
2. How long (in minutes) has it taken you to fall asleep each night? ______________
3. When have you usually gotten up in the morning? ______________
4. How many hours of actual sleep do you get at night? (This may be different than the number of hours you spend in bed) ______________
5. During the past month, how often have you had trouble sleeping because you...
a. Cannot get to sleep within 30 minutes
b. Wake up in the middle of the night or early morning
c. Have to get up to use the bathroom
d. Cannot breathe comfortably
e. Cough or snore loudly
f. Feel too cold
g. Feel too hot
h. Have bad dreams
i. Have pain
j. Other reason(s), please describe, including how often you have had trouble sleeping because of this reason(s):
6. During the past month, how often have you taken medicine (prescribed or “over the counter”) to help you sleep?
7. During the past month, how often have you had trouble staying awake while driving, eating meals, or engaging in social activity?
8. During the past month, how much of a problem has it been for you to keep up enthusiasm to get things done?
9. During the past month, how would you rate your sleep quality overall?

**Depression, Anxiety, and Stress Scale-21**^2^

Response options included: Did not apply to me at all (0), Applied to me to some degree or some of the time (1), Applied to me a considerable degree or a good part of the time (2), Applied to me very much or most of the time (3).

**Depression**

1. I couldn’t seem to experience any positive feeling at all
2. I found it difficult to work up the initiative to do things
3. I felt that I had nothing to look forward to
4. I felt down-hearted and blue
5. I was unable to become enthusiastic about anything
6. I felt I wasn’t worth much as a person
7. I felt that life was meaningless

**Anxiety**

1. I was aware of dryness of my mouth
2. I experienced breathing difficulty (e.g. excessively rapid breathing, breathlessness in the absence of physical exertion)
3. I experienced trembling (e.g. in the hands)
4. I was worried about situations in which I might panic and make a fool of myself
5. I felt I was close to panic
6. I was aware of the action of my heart in the absence of physical exertion (e.g. sense of heart rate increase, heart missing a beat)
7. I felt scared without any good reason I felt that life was meaningless

**Stress**

1. I found it hard to wind down
2. I tended to over-react to situations
3. I felt that I was using a lot of nervous energy
4. I found myself getting agitated
5. I found it difficult to relax
6. I was intolerant of anything that kept me from getting on with what I was doing
7. I felt that I was rather touchy

**Events of Discrimination Scale** ^3^

Have you ever experienced discrimination, been prevented from doing something, or been hassled or made to feel inferior in any of the following situations because of your race, ethnicity, or color?

Response options included: no (0), once (1), two or three times (2.5), four or more times (5).

1. At school?
2. Getting hired or getting a job?
3. At work?
4. Getting housing?
5. Getting medical care?
6. Getting service in a store or restaurant?
7. Getting credit, bank loans, or a mortgage?
8. On the street or in a public setting?
9. From the police or in the courts?

**Appendix B. Context Images and Supplemental Results**

**Supplementary Tables**

References

1.	Buysse, D. J., Reynolds, C. F., Monk, T. H., Berman, S. R. & Kupfer, D. J. The Pittsburgh sleep quality index: A new instrument for psychiatric practice and research. *Psychiatry Research* **28**, 193–213 (1989).

2.	Lovibond, S. H. & Lovibond, P. F. *Manual for the Depression Anxiety Stress Scales*. (Psychology Foundation, Sydney, 1995).

3.	Krieger, N., Smith, K., Naishadham, D., Hartman, C. & Barbeau, E. M. Experiences of discrimination: Validity and reliability of a self-report measure for population health research on racism and health. *Social Science & Medicine* **61**, 1576–1596 (2005).
